# Incremental Prognostic Value of Regurgitant Fraction in Patients with Ventricular Secondary Mitral Regurgitation

**DOI:** 10.3390/jcm15103854

**Published:** 2026-05-17

**Authors:** Jana Ambrožič, Dušica Prodanova, Ana Starc, Mojca Škafar, Ljupka Dimitrovska, Janez Toplišek, Mojca Bervar, Matjaž Bunc, Marta Cvijić

**Affiliations:** 1 Department of Cardiology, University Medical Centre Ljubljana, Zaloska 7, 1000 Ljubljana, Slovenia; mojca.skafar@gmail.com (M.Š.); ljupkadim@gmail.com (L.D.); jtoplisek@gmail.com (J.T.); bervarmojca@gmail.com (M.B.); mbuncek@yahoo.com (M.B.); marta.cvijic@gmail.com (M.C.); 2 Faculty of Medicine, University of Ljubljana, Vrazov trg 2, 1000 Ljubljana, Slovenia; dusicaprodanova@yahoo.co.uk (D.P.); starc.anastarc.ana@gmail.com (A.S.)

**Keywords:** mitral regurgitation, quantification, PISA method, regurgitant fraction, outcome

## Abstract

**Objectives:** Quantifying ventricular secondary mitral regurgitation (MR) remains challenging, and the prognostic value of echocardiographic parameters is uncertain. This study aimed to assess the concordance of parameters of MR severity and determine the added value of regurgitant fraction (RF) in predicting outcomes. **Methods and results:** We retrospectively analysed 186 patients with ventricular secondary MR who underwent echocardiography with MR assessment, evaluating effective regurgitant orifice area (EROA), regurgitant volume (RegVol) and RF. The primary endpoint was a composite of all-cause death or heart failure hospitalisation. Quantitative parameters of MR severity were frequently discordant. Using the guideline-recommended cut-offs for EROA (≥40 mm^2^), RegVol (≥60 mL) and RF (≥50%), severe MR was present in 5.4%, 3.3%, and 29.5% of patients, respectively. Both RF ≥ 50% and EROA ≥ 40 mm^2^ were independently associated with clinical outcomes in multivariable Cox models. Combining RF and EROA provided incremental prognostic value over either parameter alone (*p* < 0.05). Kaplan–Meier curves showed that patients with EROA < 40 mm^2^ and RF ≥ 50% had similar outcomes to those with EROA ≥ 40 mm^2^ (*p* = 0.055), whereas patients with both EROA < 40 mm^2^ and RF < 50% had significantly better outcomes (*p* = 0.002). **Conclusions:** Substantial discordance between quantitative parameters of severe MR was observed in ventricular secondary MR. RF is a strong, underutilised marker of MR severity, reflecting haemodynamic burden beyond EROA and RegVol. Patients with EROA < 40 mm^2^ and RF > 50% had outcomes comparable to those who met the guideline-based threshold for severe MR, defined as EROA ≥ 40 mm^2^. Our results demonstrate that routine RF assessment may enhance risk stratification and enable identification of a high-risk subgroup of patients with EROA < 40 mm^2^.

## 1. Introduction

Accurate quantification of the severity of ventricular secondary mitral regurgitation (MR) is essential for risk stratification and clinical decision-making regarding the timing of interventions. Several parameters have been described for quantifying MR, including effective regurgitant orifice area (EROA), regurgitant volume (RegVol) and regurgitant fraction (RF). Previous studies have demonstrated the prognostic value of EROA in patients with secondary MR [1,2,3,4,5]. These studies have shown that the risk of all-cause death and/or heart failure hospitalisation increases above certain thresholds, ranging from 6 to 30 mm^2^. These diverging thresholds introduce uncertainty regarding a uniform cut-off value for EROA across the entire spectrum of patients with secondary MR. As shown in the theoretical paper, EROA and RegVol values associated with severe secondary MR depend not only on haemodynamic status, but also on left ventricular (LV) size and ejection fraction [6]. Therefore, defining the magnitude of valve dysfunction purely based on lesion severity assessment by EROA or RegVol might be problematic, as using uniform cut-offs could lead to underestimation of the haemodynamic significance of valve lesions in specific patient groups.

Conversely, RF, which was rarely analysed in previous publications, provides a size-independent measure of MR severity and defines volume overload of the valve lesion. An RF greater than 50% appears to be a reasonable cut-off for defining haemodynamic significance, as more than half of the total LV stroke volume is lost backwards into the left atrium. RF might provide a better definition for the haemodynamic significance of MR than EROA or RegVol, as it already accounts for low flow conditions. There are limited data suggesting that using RF improves risk stratification of patients with secondary MR [7], but further research is needed to confirm this.

Accordingly, the aim of our study was to (1) evaluate the concordance of quantitative parameters of MR severity, (2) assess the prognostic value of individual quantitative parameters of MR severity, and (3) determine the added value of RF over traditional quantitative parameters in patients with ventricular secondary MR.

## 2. Materials and Methods

### 2.1. Study Population

A retrospective study was conducted among patients who were referred for clinically indicated echocardiography between 1 January and 31 December 2019, at the Department of Cardiology, University Medical Centre Ljubljana. The study flow chart is shown in Figure S1. Briefly, consecutive patients with at least mild secondary MR using a multiparametric integrative approach and history of LV systolic dysfunction caused by either dilated or ischemic cardiomyopathies, as well as those who had basal inferior myocardial infarction resulting in posterior leaflet tethering, were included. Patients with MR, in whom the proximal isovelocity surface area (PISA) method for MR quantification could not be performed—such as those with eccentric regurgitant jets, multiple jets, or no visible PISA radius—were excluded. Additional exclusion criteria were more than mild aortic regurgitation, mitral stenosis or previous mitral valve replacement. Echocardiograms were reviewed from the echocardiographic database, and clinical data were obtained from medical records. The study protocol was approved by the National Medical Ethics Committee of the Republic of Slovenia.

### 2.2. Echocardiographic Assessment

Standard 2D and Doppler transthoracic echocardiography was performed using commercial echocardiographic machines (Vivid E9, Vivid S60 (GE Vingmed Ultrasound AS, Horten, Norway), iE33 (Philips Medical Systems, Bothell, WA, USA)). Digitally stored data were retrospectively analysed offline (Echopac, version 20.4, GE Vingmed Ultrasound AS). The standard measurements were performed in accordance with the most recent recommendations [8]. LV volumes and ejection fraction (LVEF) were estimated by the Simpson’s biplane method. LV total stroke volume (total SV) was calculated as the difference between LV end-diastolic (LVEDV) and end-systolic volume (LVESV). Forward SV was calculated from the velocity time integral (VTI) of the LV outflow tract measured by the pulsed wave Doppler, multiplied by the LV outflow tract area [9,10]. Conventional parameters for right ventricle (RV) size and function, as well as pulmonary artery systolic pressure, were measured according to the recommendations [11]. The integrative approach for assessing MR severity was used for baseline classification at study inclusion; however, prognostic analyses were based on individual quantitative PISA parameters, which were analysed separately. The following quantitative parameters of MR severity were reported: EROA, RegVol and RF by the PISA method (Graphical abstract). EROA and RegVol were calculated by the standard PISA approach using a measurement of PISA radius at mid-systole [9,10]. RF was calculated as the percentage of RegVol to total SV.

### 2.3. Outcome

All patients were followed until the end of 2023. The study outcome was a composite of all-cause death or hospitalisation due to heart failure. Patients were censored at the time of mitral valve intervention or at the end of follow-up if they did not experience the event. Follow-up data were obtained by retrospective review of hospital medical records and the national health database. Physicians, blinded to echocardiographic data, assigned clinical events.

### 2.4. Statistical Analysis

Continuous variables were presented as mean ± standard deviation, if normally distributed, otherwise as medians and interquartile ranges. Categorical data were summarised as frequencies and percentages. The unpaired Student *t*-test or Mann–Whitney test was used to compare two groups. Categorical variables were compared using a chi-square test or Fisher’s exact test. Associations between parameters of MR severity were assessed using Pearson correlation coefficients, while Cohen’s Kappa coefficients evaluated concordance between guideline-recommended cut-offs. Kaplan–Meier curve analysis was performed to estimate event-free survival rates, and log-rank tests were used to assess differences between groups. To assess the pattern of MR severity parameters associated with clinical outcome, a restricted spline curve with four knots was used, where the x-axis represents MR severity parameters as a continuous variable, and the y-axis represents the unadjusted hazard ratio (HR) for the composite endpoint. Univariate and multivariate Cox proportional hazards models were used to assess association with survival, presented as HR with corresponding 95% confidence intervals (CI). Clinically relevant variables and those showing significant association in univariable analyses were entered into a multivariate model. A rule of 10 events per variable was applied to prevent overfitting of the multivariate model [12]. Collinearity of variables was tested using the variance inflation factor (considered excessive if >3) and the variance proportion (considered excessive if >0.5). LVESV, LVESV index (LVESVi) and LVEF, tricuspid annular plane systolic excursion (TAPSE) and RV diameter, RegVol and EROA were not included together in the multivariate analysis due to strong collinearity. To compare the prognostic value of MR severity parameters, different multivariable models were constructed using the same baseline model. The baseline model was the best-fit model using clinical variables and echocardiographic parameters of LV and RV size/function (age, LVESV index, SV index, RV diameter and pulmonary systolic pressure). The predictive accuracy (discriminative ability) of different models was assessed using likelihood ratio tests to evaluate changes in χ^2^ values. Intraobserver and interobserver reproducibility of the MR parameters were tested in 20 randomly selected patients using the intraclass correlation coefficient (ICC). A two-tailed *p*-value of ≤0.05 was considered statistically significant. Statistical analyses were performed using SPSS version 20.0 (IBM, Chicago, IL, USA) and R version 4.4.2 (Vienna, Austria).

## 3. Results

### 3.1. Study Population

The study cohort included 186 patients with ventricular secondary MR. Of these, 19 (10.2%) patients had MR grade IV based on the integrative approach, 14 (7.5%) had grade III, 91 (48.9%) had grade II, and 62 (33.3%) had grade I. Approximately half of the patients had ischaemic cardiomyopathy, and nearly a third had atrial fibrillation. Other clinical and echocardiographic data are listed in Table 1. The proportions of patients treated with guideline-directed medical therapy were similar at baseline and at the last follow-up.

### 3.2. Comparison of Parameters of MR Severity

**Figure 1:**
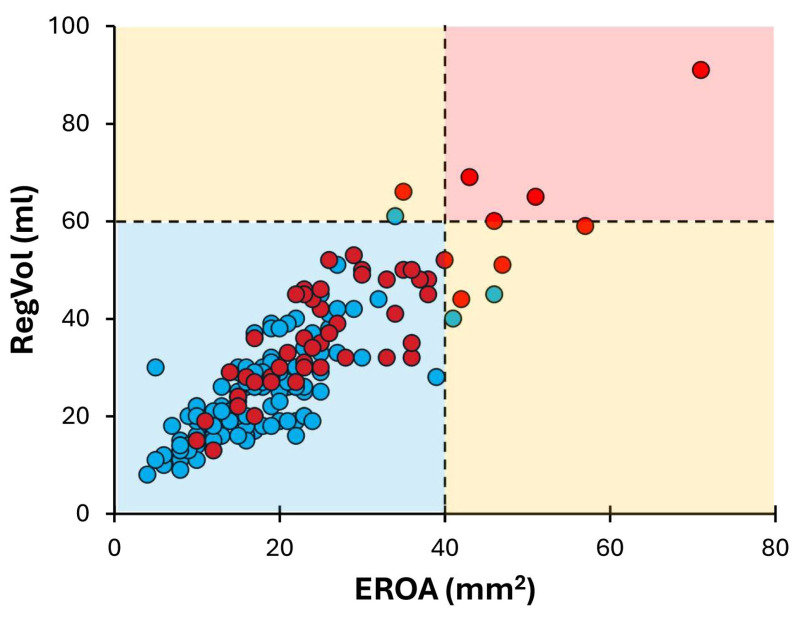
Correlation and concordance among the parameters of MR severity. EROA and RegVol correlated well with each other (r = 0.855, *p* < 0.001). The red box indicates patients with concordant severe MR, and the blue box indicates patients with concordant non-severe MR, while yellow boxes represent patients with discordant MR severity, when using the recommended cut-offs: EROA (≥40 mm^2^) or RegVol (≥60 mL). Red dots represent patients with RF ≥ 50%, while blue dots represent patients with RF < 50%. A substantial number of patients with RF > 50% (red dots) were classified as non-severe MR according to EROA or RegVol cut-offs (blue box). Abbreviations as in Graphical abstract.

All quantitative parameters for MR severity correlated well with each other, although not perfectly (EROA vs. RegVol: r = 0.855, *p* < 0.001; EROA vs. RF: r = 0.586, *p* < 0.001; RegVol vs. RF: r = 0.584, *p* < 0.001). The correlation and concordance of the quantitative measurements are shown in the Graphical abstract and Figure 1. Using the guideline-recommended cut-offs for EROA (≥40 mm^2^), RegVol (≥60 mL) and RF (≥50%), severe MR was present in 10 (5.4%), 6 (3.3%) and 54 (29.5%) patients, respectively. The observed agreement between the recommended cut-offs was 96% for EROA and RegVol, with a Kappa coefficient of 0.479 (95% CI 0.172–0.786, *p* < 0.001). The agreement between the recommended cut-offs was lower for EROA and RF (observed agreement 74%; Kappa coefficient: 0.174 (95% CI 0.051–0.296, *p* < 0.001)) and for RegVol and RF (observed agreement 73%; Kappa coefficient: 0.114 (95% 0.011–0.218), *p* < 0.001). Patients with discordant grading (EROA vs. RF) had smaller LV and lower SV than those with concordant grading (Table S1).

Intra- and interobserver variability was good for all MR measurements (EROA: intraobserver: ICC 0.97, 95% CI [0.92–0.99]; interobserver: ICC 0.96 [0.92–0.98]; RegVol: intraobserver: ICC 0.96 95% CI [0.91–0.99]; interobserver: ICC 0.96 [0.90–0.99]; RF: intraobserver: ICC 0.94, 95% CI [0.85–0.97]; interobserver: ICC 0.92 [0.83–0.97]).

### 3.3. Association of MR Severity with Clinical Outcomes

During a median follow-up of 769 (106–1584) days, 107 patients reached the composite endpoint (50 deaths and 57 hospitalisations due to heart failure). Approximately 7% (13) of patients underwent mitral valve intervention (baseline characteristics of this cohort presented in Table S2). Patients who reached the composite endpoint were older and had more pronounced LV and RV remodelling, as well as more impaired systolic function (Table 1). At baseline, the proportion of patients using diuretics was higher in the group with the composite endpoint. During follow-up, there was no significant change in the proportion of medication use in any of the groups. Regarding the quantitative parameters of MR severity, patients with clinical events had higher RF, while EROA and RegVol were comparable.

**Figure 2:**
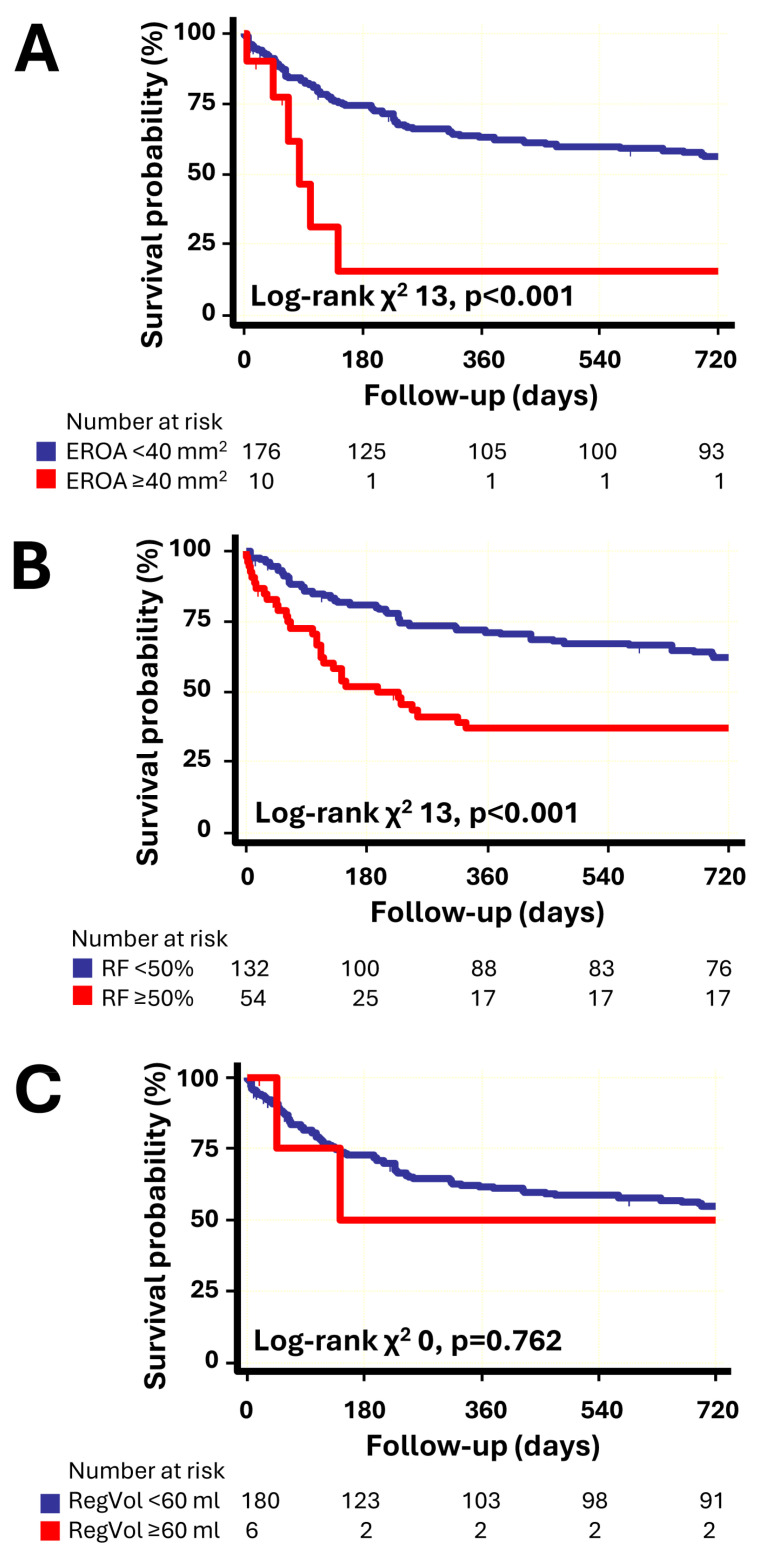
Kaplan–Meier curve analysis of event-free survival according to parameters of MR severity: (**A**) EROA, (**B**) RF, (**C**) RegVol. Due to the small sample size in the group with EROA ≥ 40 mm^2^ and RegVol ≥ 60 mL, the results should be interpreted with caution and should not be considered as evidence of absent prognostic value for this parameter. Number of outcome events: EROA ≥ 40 mm^2^: 7 patients, RF ≥ 50%: 37 patients, and RegVol ≥ 60 mL: two patients. Abbreviations as in Graphical abstract.

Kaplan–Meier analysis for composite clinical outcome showed that the recommended cut-off values for EROA and RF significantly discriminated cumulative survival between patients with severe and non-severe MR, while no significant differences were observed in survival when assessing severity with RegVol (Figure 2).

**Figure 3:**
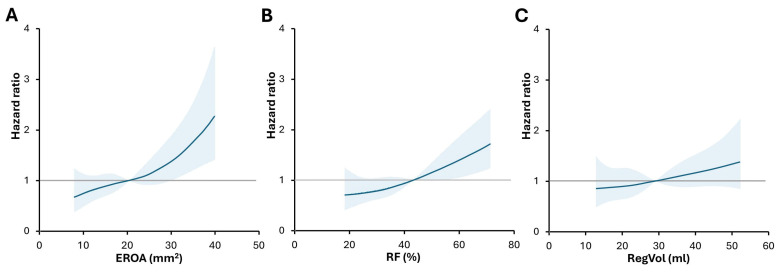
Spline curve showing the association between (**A**) EROA, (**B**) RF, (**C**) RegVol and outcome. The HR of 1 represents the mean risk of the study cohort. Solid blue line indicates HR, while the shaded area indicates 95% confidence interval. An excess risk for events (HR > 1) is observed at around 20 mm^2^ for EROA, 45% for RF and 30 mL for RegVol. HR—hazard ratio, other abbreviations as in Graphical abstract.

To assess the continuous association between quantitative parameters of MR severity and hazard ratio for the clinical endpoint in our study cohort, spline curve analysis was performed. The risk for the composite endpoint began to increase at levels below the recommended cut-off for severe MR. Excess risk (HR > 1) was observed at around 20 mm^2^ for EROA, 45% for RF and 30 mL for RegVol (Figure 3).

The parameters associated with the composite endpoint in the univariate Cox regression analysis are presented in Table 2. In univariate analysis, EROA ≥ 40 mm^2^ and RF ≥ 50% were strongly associated with the composite endpoint, while RegVol ≥ 60 mL did not show a significant association. In multivariable analysis, the association of EROA ≥ 40 mm^2^, RegVol ≥ 60 mL and RF ≥ 50% with the composite endpoint was tested in addition to clinical and echocardiographic variables, including age, LVESV index, SV index, RV diameter and pulmonary systolic pressure (baseline model) (Table 3). Both EROA ≥ 40 mm^2^ and RF ≥ 50% were independently associated with increased HR for the composite endpoint. Additionally, no significant interaction was observed between RF and LVEF subgroups (*p* for interaction 0.419), suggesting a consistent prognostic effect of RF across LVEF subgroups (Figure S2).

Using quantitative parameters of MR severity as continuous variables demonstrated similar results (Tables S3 and S4).

### 3.4. Added Value of Combining EROA and RF for the Composite Endpoint

**Figure 4:**
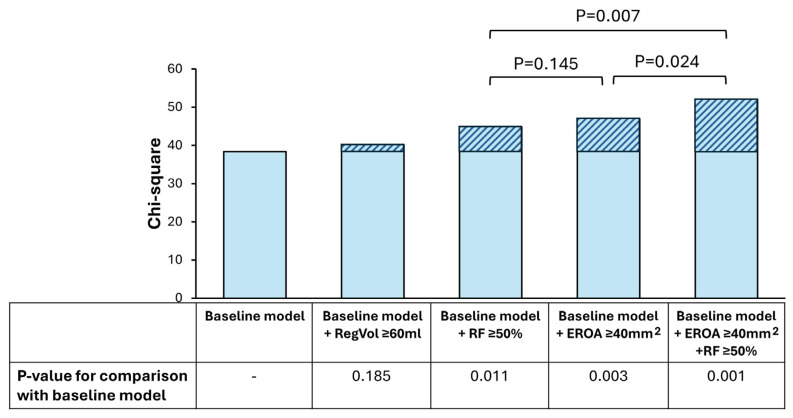
Prognostic value of quantitative parameters of MR severity. Baseline model includes age, left ventricular end-systolic volume index, stroke volume index, right ventricular diameter and pulmonary systolic pressure. Models with RF ≥ 50% and EROA ≥ 40 mm^2^ were more accurate than baseline model in predicting risk of clinical endpoints, while there was no significant difference in prognostic power between them (*p* = 0.145). The model combining RF ≥ 50% and EROA ≥ 40 mm^2^ provides incremental prognostic value over models using single parameter of MR severity. The bar graphs show the chi-squares values for each model; the marked section of each bar indicates the change in chi-squares compared to the baseline model. Abbreviations as in Graphical abstract.

As shown in Figure 4, adding either EROA ≥ 40 mm^2^ or RF ≥ 50% to the baseline model (age, LVESV index, SV index, RV diameter and pulmonary systolic pressure) improved the model and its association with clinical outcome. There was no significant difference in the predictive value of the model containing EROA or RF (*p* = 0.145). Furthermore, the model including both EROA and RF significantly outperformed models using either EROA ≥ 40 mm^2^ or RF ≥ 50% alone, demonstrating the increased prognostic value of combining EROA and RF to assess clinical outcome.

In the analysis, using MR severity parameters as continuous variables, the model with RF showed the strongest predictive value for the composite outcome, while adding EROA to the model with RF did not provide any further significant incremental value (Figure S3).

**Figure 5:**
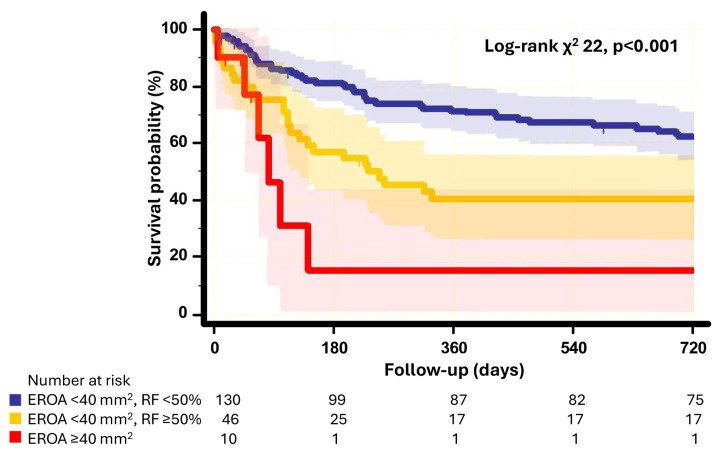
Kaplan–Meier curve analysis of event-free survival according to EROA and RF values. Comparison of survival between 3 patient subgroups: EROA ≥ 40 mm^2^ vs. EROA < 40 mm^2^ + RF ≥ 50% vs. EROA < 40 mm^2^ + RF < 50%. Shading represents 95% confidence interval. Abbreviations as in Graphical abstract.

As EROA is typically used as a basic parameter to assess MR severity, we further evaluated risk stratification using RF in a patient group with EROA < 40 mm^2^ (Graphical abstract). Kaplan–Meier analysis showed that RF can be used for risk re-stratification in patients with EROA < 40 mm^2^ (Figure 5). Patient subgroup with both EROA < 40 mm^2^ and RF < 50% had comparatively lower event rates for composite endpoints than patients with both EROA < 40 mm^2^ and RF ≥ 50% (log rank *p* = 0.002). In contrast, the patient group with both EROA < 40 mm^2^ and RF ≥ 50% and the group with EROA ≥ 40 mm^2^ showed similar event rates (log rank *p* = 0.055).

## 4. Discussion

The key findings of our study exploring the prognostic value of quantitative parameters of MR severity in patients with ventricular secondary MR are as follows:

(i) substantial discordance in MR grading was observed between EROA and RF, as well as between RegVol and RF,
(ii) both EROA and RF were independently associated with clinical outcome, whereas no such association was found for RegVol,
(iii) increased risk for the composite endpoint was observed at lower thresholds of quantitative parameters than recommended cut-off values for severe ventricular secondary MR, and
(iv) the combination of EROA, as a descriptor of lesion severity, and RF, as a descriptor of the haemodynamic significance of volume overload, provided incremental prognostic value over single parameters of MR severity. Importantly, RF demonstrated added value for further risk stratification in patients with non-severe MR according to EROA.

### 4.1. Concordance of Quantitative Parameters of MR Severity

Our results showed a good correlation between quantitative parameters of MR severity but marked discordance in the definition of severe MR between EROA and RF, and between RegVol and RF (in 26% and 27% of patients, respectively), whereas discordance between EROA and RegVol was relatively rare (4%). Previous studies have also found limited agreement on the quantitative parameters of MR severity [7,13,14]. Only 27% of patients in the predefined intermediate risk group had concordant parameters of EROA and RF [7]. Additionally, we showed that these discordances were more frequent in patients with smaller LV volumes and lower SV, consistent with the notion that EROA and RegVol are susceptible to underestimating the haemodynamic severity of MR in low flow conditions, as previously described [5,6].

Accurate assessment of the severity of MR remains challenging, particularly in the setting of underlying LV dysfunction, low flow conditions and specific characteristics of the regurgitant orifice [9,10]. Grading MR severity using EROA or RegVol by the PISA method may underestimate MR due to the crescent-shaped regurgitant orifice and the dynamic pattern of regurgitant flow [10,14,15,16]. Additionally, pressure gradients and orifice calculations are highly flow dependent. By contrast, RF considers the total volume of blood ejected by the LV and accounts for systemic flow. Therefore, EROA, RegVol and RF may show discordance in low or high flow states and may vary with LV size and function, as well as the pressure gradient between the LV and left atrium [6,10]. For example, a RegVol of 30 mL and EROA of 20 mm^2^ may be associated with RF ≥ 50% in a patient with reduced LVEF and reduced total LV stroke volume [6]. Therefore, RF may provide complementary measures regarding the haemodynamic significance of MR, particularly in low flow states, where EROA and RegVol may underestimate the regurgitant load (Graphical abstract). Accordingly, current guidelines [17,18,19] have recognised that the accepted thresholds for defining severe MR may be lower (EROA ≥ 30 mm^2^), based on low flow conditions and the above-mentioned PISA considerations. It is worth noting that the definition of low flow state commonly used in the literature is based on an SV index < 35 mL/m^2^ and has mainly been used to classify low flow low gradient aortic stenosis [20]. Current guidelines do not provide specific recommendations for defining or adjusting for low flow states in MR [17,18,19]. Further studies are needed to determine which approach should be taken when measures of MR severity are discordant, and to better define haemodynamic states and echocardiographic characteristics when lower thresholds of EROA might be applicable.

### 4.2. Association of MR Grading with Clinical Outcome

The causal relationship between secondary MR and clinical outcome remains controversial [1,2,4,5,7,21,22,23,24]. Several studies have reported an association between parameters of MR severity and increased risk of adverse events in patients with reduced LVEF [2,3,5,7]. In our study, both EROA and RF, besides the parameters of LV size and flow condition, were independently associated with adverse outcomes, reinforcing their individual prognostic value, whereas no significant association was found for RegVol. However, due to the limited sample size of the RegVol ≥ 60 mL group, that analysis was insufficiently powered to reliably assess the prognostic impact of RegVol. A unique finding of our study is that the combination of EROA and RF provided the highest predictive accuracy for the composite endpoint, suggesting that including both parameters improves risk stratification compared to either parameter alone. These findings suggest that RF, as a flow-normalised parameter, may offer additional prognostic value by reflecting the haemodynamic burden of MR, in addition to the commonly used EROA.

Our results indicate that, in accordance with physiological concepts, it is important to consider both the valve lesion and the haemodynamic significance of volume overload of MR. As previously mentioned, EROA and RegVol thresholds that define severe MR are related to LV volumes, LVEF and mean systolic pressure gradient between the LV and left atrium [6], while RF provides a normalised measure of RegVol to total stroke volume, enabling direct comparisons across patients with different LV sizes and haemodynamic states. This is particularly valuable in secondary MR, where the valve lesion, as assessed by EROA, can be within the range of moderate MR, but constitutes an RF > 50%, consistent with haemodynamically severe MR [6]. It appears that RF, while underutilised in everyday clinical practice, may provide a more volume-adjusted measure of MR severity and offer clinically meaningful added value to other parameters of MR severity.

It is well recognised that an EROA ≥ 40 mm^2^ identifies patients with haemodynamically severe secondary MR and worse outcomes, while patients with EROA < 40 mm^2^ represent a highly heterogeneous cohort with varying outcomes. Patients with discordant grading—EROA < 40 mm^2^ and RF ≥ 50%—constituted a high-risk subgroup, as their survival rate was similar to that of patients with severe MR defined by EROA ≥ 40 mm^2^. However, these findings should be interpreted cautiously, given the limited number of patients with EROA ≥ 40 mm^2^. Our data corroborate a previous study, which observed a threefold increase in mortality risk in the intermediate-risk subgroup of patients with non-severe MR (EROA 20–29 mm^2^ and RegVol 30–44 mL) and with RF ≥ 50%, compared to patients with RF < 50% [7].

These findings may have implications for patient selection in the contemporary era of transcatheter interventions. Randomised trials of transcatheter-edge-to-edge repair in secondary MR, including COAPT, MITRA-FR and more recently RESHAPE-HF2, have highlighted the marked heterogeneity of secondary MR and underscored the importance of patient selection in the context of the interplay between MR severity and LV size and function [25]. As such, RF may provide additional value by identifying patients with apparently non-severe MR according to EROA, but with significant regurgitant load and adverse prognosis. Our findings support the concept that some patients with moderate MR but RF ≥ 50% may behave clinically as severe MR patients and could potentially benefit from intervention. Whether RF-guided stratification may improve the selection of patients for intervention requires further studies.

There is substantial data on specific thresholds of quantitative parameters associated with worse outcomes in secondary MR, with particular focus on EROA. Some data showed that categorical presence of secondary MR was associated with increased risk, but there was no linear increase in mortality risk with increasing EROA above zero [23], while another study demonstrated that the rate of excess mortality was stable up to EROA of 10 mm^2^, then increased exponentially with rising EROA without plateau [5]. Our spline curve analysis showed a continuous increase in risk with increasing MR severity parameters, with an excess of adverse events (HR > 1) at around EROA 20 mm^2^, RegVol 30 mL and RF 45%. Other authors have also demonstrated that the risk begins to increase at EROA or RegVol levels below the guideline-recommended cut-offs for severe MR [3,4,5,7]. Studies have reported different thresholds at which significantly higher risk occurs, ranging from 10 mm^2^ to 30 mm^2^ for EROA and 30 mL to 60 mL for RegVol. The diversity of reported thresholds might be due to heterogeneous study cohorts, including both entities of secondary MR—atrial [4,23] and ventricular MR [3,5,7]—as well as a broad spectrum of LV size and systolic function. This highlights the potential limitation of a uniform threshold for EROA and RegVol, given their important, yet often underappreciated dependence on LV pressure, volume and LVEF [6,26]. Conversely, outcome data regarding specific RF thresholds in secondary MR are lacking [4,7]. Similar to our results, studies identify a threshold for significantly higher risk at around RF 50%, despite very diverse study cohorts. In contrast to EROA and RegVol, the prognostic value of RF aligns perfectly with the guideline definition of severe MR. These results once again raise an intriguing debate about whether we should base the criteria for quantifying ventricular secondary MR on prognostic information.

### 4.3. Clinical Implications

The main finding of our study is the incremental value of RF in refining risk stratification and guiding more individualised clinical decisions, especially when quantitative parameters are discordant. The combination of EROA as a descriptor of lesion severity and RF as a descriptor of the haemodynamic burden of MR may improve risk stratification compared to either parameter alone, and as such may be a valuable clinical tool in decision making.

Our data support a greater emphasis on quantitative metrics, especially RF, when predicting patient outcomes. Additionally, it should be recognised that an EROA < 40 mm^2^ does not necessarily imply a favourable prognosis, and that flow conditions, LV size and function, as well as haemodynamic significance of volume overload as assessed by RF, should be considered.

Our findings do not support replacing the current guideline-recommended integrative approach. Rather, we suggest that RF might serve as an adjunct parameter to EROA classification and could provide additional information for the assessment and risk stratification of ventricular secondary MR (Graphical abstract). This is particularly relevant in low flow states, rendering absolute values of EROA and RegVol less reliable indicators of MR severity. In this context, RF expressed as a percentage of total SV provides a more robust index of regurgitant burden and showed consistent prognostic value in the analytic model. However, it remains to be prospectively validated whether this concept can be used to improve patient selection for valve interventions and consequently reduce mortality and heart failure hospitalisation.

### 4.4. Limitations

Our study is retrospective and single-centred with a relatively small number of patients, which may limit its generalisability. The population was restricted to ventricular secondary MR, so the results may not apply to primary MR or atrial secondary MR. Some patients in our study group might have dual functional MR. However, according to our inclusion criteria, patients primarily met the criteria for ventricular MR, meaning that the atrial component could not be the leading mechanism of MR. The interpretation of survival analyses in the subgroups of EROA ≥ 40 mm^2^ and RegVol ≥ 60 mL should be made cautiously due to the limited number of patients in these categories. Therefore, these findings should be considered exploratory, given the sparse data and limited statistical power.

The use of single-frame mid-systolic PISA measurements, although consistent with recommendations, may underestimate the severity of regurgitation in dynamic MR. Additionally, we used Simpson’s method instead of the Doppler method with inflow VTI at the mitral annulus to assess total SV. The Doppler method uses a greater number of potential variables, such as mitral annulus geometry, sample volume position and angulation, that can affect the precision of the SV calculation compared to the Simpson’s method. SV measured by the Simpson’s method is an accurate measurement, which showed a good correlation with similar measurements by cardiac magnetic resonance (CMR) [27].

Finally, advanced imaging modalities such as 3D echocardiography (e.g., 3D vena contracta area) or CMR might provide more robust quantification and risk stratification in future studies. A recent study has already demonstrated that EROA measured by the 3D echocardiographic volumetric method improves risk stratification in patients with ventricular MR compared to the standard PISA method [3].

## 5. Conclusions

Our study highlights the challenges and new insights associated with quantifying ventricular secondary MR. Substantial discordance between quantitative parameters of severe MR was frequently observed in ventricular secondary MR in routine clinical practice. Increased risk of adverse events appeared at EROA of around 20 mm^2^ with a continuous increase as EROA increased. Among patients with EROA < 40 mm^2^, those with RF ≥ 50% had worse outcomes, suggesting that RF may help identify a high-risk subgroup of patients with non-severe MR according to EROA. These findings may underscore the clinical value of routinely assessing RF to refine risk stratification and identify a high-risk subgroup of patients with EROA < 40 mm^2^. Integrating RF assessment could improve diagnostic precision and risk stratification in patients with ventricular secondary MR and might potentially complement existing grading approaches. Overall, these results should be considered hypothesis-generating. Further prospective studies are needed to validate these findings and to explore their potential integration into clinical decision-making for patients with ventricular secondary MR.

## Figures and Tables

**Table 1:** Clinical and echocardiographic characteristics of the study population according to the composite clinical endpoint.

	All Patients (*n* = 186)	No Events (*n* = 79)	Events (*n* = 107)	*p*-Value
Clinical characteristics				
Age (years)	69 ± 12	65 ± 13	72 ± 10	0.001
Gender (male)	129 (69)	56 (71)	73 (68)	0.697
BMI (kg/m^2^)	27 ± 5	28 ± 4	27 ± 5	0.299
Systolic blood pressure (mmHg)	122 ± 20	125 ± 19	119 ± 20	0.106
Heart rate (bpm)	77 ± 19	76 ± 22	77 ± 17	0.700
Atrial fibrillation (%)	50 (27)	18 (23)	32 (27)	0.279
Ischemic cardiomyopathy (%)	101 (54)	41 (52)	60 (56)	0.572
Echocardiographic characteristics				
LVEDD (mm)	62 ± 10	62 ± 9	62 ± 11	0.990
LVESD (mm)	51 ± 12	50 ± 11	51 ± 12	0.414
LVEDV index (mL)	100 (79–124)	95 (77–119)	107 (83–126)	0.109
LVESV index (mL)	64 (44–84)	58 (36–76)	69 (49–88)	0.017
LVEF (%)	35 (29–45)	41 (32–42)	34 (27–42)	0.001
Stroke volume index (mL/m^2^)	31 ± 11	33 ± 13	29 ± 8	0.028
CO (L/min)	4.2 (3.4–5.0)	4.1 (3.4–5.4)	4.2 (3.2–4.8)	0.199
RV basal diameter (mm)	41 ± 8	39 ± 7	42 ± 8	0.001
TAPSE (mm)	18 ± 5	19 ± 5	18 ± 4	0.009
PASP (mmHg)	48 ± 13	46 ± 14	50 ± 12	0.068
EROA (mm^2^)	19 (14–25)	17 (12–23)	19 (14–25)	0.140
RegVol (mL)	27 (19–37)	26 (20–33)	27 (19–37)	0.640
RF (%)	39 (29–54)	36 (25–46)	40 (30–59)	0.017
Medications				
RAAS inhibitor or ARNI (%)	169 (91)	72 (91)	97 (91)	0.910
Beta-blocker (%)	160 (90)	71 (90)	89 (83)	0.193
MRA (%)	124 (67)	50 (63)	74 (69)	0.401
Diuretic (%)	146 (79)	48 (61)	97 (90)	<0.001

Legend: ARNI—angiotensin receptor neprilysin inhibitor, BMI—body mass index, EROA—effective regurgitant orifice area, LVEDD—left ventricular end-diastolic diameter, LVEDV—left ventricular end-diastolic volume, LVEF—left ventricular ejection fraction, LVESD—left ventricular end-systolic diameter, LVESV—left ventricular end-systolic volume, MRA—mineralocorticoid receptor antagonist, PASP—pulmonary artery systolic pressure, RAAS—renin–angiotensin–aldosterone system (including angiotensin-converting enzyme inhibitor and angiotensin receptor blocker), RegVol—regurgitant volume, RF—regurgitant fraction, RV—right ventricle, TAPSE—tricuspid annular plane systolic excursion.

**Table 2:** Univariate Cox regression analysis for the composite endpoint.

	HR (95% CI)	*p*-Value
Age (years)	1.032 (1.016–1.050)	<0.001
Male sex	0.985 (0.655–1.450)	0.941
BMI (kg/m^2^)	0.962 (0.919–1.007)	0.099
Heart rate (bpm)	1.004 (0.996–1.013)	0.330
Atrial fibrillation (%)	1.236 (0.817–1.870)	0.317
Ischemic cardiomyopathy (%)	1.183 (0.807–1.734)	0.389
LVEDD (mm)	1.004 (0.984–1.024)	0.706
LVESD (mm)	1.011 (0.994–1.029)	0.199
LVEDV index (mL)	1.004 (0.999–1.008)	0.098
LVESV index (mL)	1.007 (1.002–1.011)	0.007
LVEF (%)	0.967 (0.951–0.984)	<0.001
Stroke volume index (mL/m^2^)	0.976 (0.957–0.995)	0.012
RV basal (mm)	1.768 (1.349–2.317)	<0.001
TAPSE (mm)	0.520 (0.338–0.800)	0.003
PASP (mmHg)	1.024 (1.009–1.039)	0.001
EROA (≥40 mm^2^)	3.794 (1.728–8.330)	0.001
RegVol (≥60 mL)	0.806 (0.199–3.268)	0.762
RF (≥50%)	2.073 (1.385–3.103)	<0.001

Legend: abbreviation as in Table 1.

**Table 3:** Multivariable Cox regression models for the composite clinical endpoint.

	Model 1		Model 2		Model 3	
	HR (95% CI)	*p*-Value	HR (95% CI)	*p*-Value	HR (95% CI)	*p*-Value
Age (years)	1.036 (1.015–1.058)	0.001	1.041 (1.020–1.063)	<0.001	1.037 (1.016–1.059)	0.001
LVESV index (mL)	1.007 (1.001–1.012)	0.016	1.006 (1.000–1.011)	0.049	1.008 (1.002–1.013)	0.006
SV index (mL/m^2^)	0.975 (0.954–0.996)	0.022	0.976 (0.955–0.998)	0.033	0.978 (0.957–0.999)	0.039
RV basal (mm)	1.627 (1.197–2.212)	0.002	1.577 (1.158–2.147)	0.004	1.592 (1.184–2.142)	0.002
PASP (mmHg)	1.007 (0.990–1.025)	0.401	1.005 (0.988–1.022)	0.576	1.004 (0.987–1.021)	0.643
RegVol (≥60 mL)	0.523 (0.121–2.255)	0.385				
EROA (≥40 mm^2^)			2.811 (1.211–6.526)	0.016		
RF (≥50%)					1.757 (1.141–2.704)	0.010

Legend: abbreviation as in Table 1.

## Data Availability

The original contributions presented in the study are included in the article; further inquiries can be directed to the corresponding authors.

## Supplementary Materials

The following supporting information can be downloaded at: https://www.mdpi.com/article/10.3390/jcm15103854/s1, Supplemental Table S1: Characteristics of the study group with concordant grading of MR and study group with discordant grading of MR; Supplemental Table S2: Characteristics of the study group who had intervention on mitral valve; Supplemental Table S3: Univariate Cox regression analysis for the composite end point; Supplemental Table S4: Multivariable Cox regression models for the composite clinical endpoint; Supplemental Figure S1: Study flow chart; Supplemental Figure S2: Forest plot showing hazard ratio (HR) and 95% confidence interval (CI) for composite outcome in our study group; Supplemental Figure S3: Prognostic value of quantitative parameters of MR severity, as a continuous variable.

## Abbreviations

The following abbreviations are used in this manuscript:

CI	Confidence intervals
EROA	Effective regurgitant orifice area
HR	Hazard ratio
ICC	Intraclass correlation coefficient
LV	Left ventricular
LVEDV	Left ventricular end-diastolic
LVEF	Left ventricular ejection fraction
LVESV	Left ventricular end-systolic volume
LVESVi	Left ventricular end-systolic volume index
MR	Mitral regurgitation
PISA	Proximal isovelocity surface area
RegVol	Regurgitant volume
RF	Regurgitant fraction
RV	Right ventricle
SV	Stroke volume
TAPSE	Tricuspid annular plane systolic excursion
VTI	Velocity time integral
