# Supplementary material for: Incremental Prognostic Value of Regurgitant Fraction in Patients with Ventricular Secondary Mitral Regurgitation
Source: J Clin Med. 2026 May 17;15(10):3854. doi: 10.3390/jcm15103854 (PMC13206879; doi:10.3390/jcm15103854)
Supplement: Supplementary file 1 [file jcm-15-03854-s001.zip › jcm-4246195-supplementary.pdf]

SUPPLEMENTARY MATERIAL

Incremental prognostic value of regurgitant fraction in patients with ventricular secondary mitral regurgitation

Jana Ambrožič, Dušica Prodanova, Ana Starc, Mojca Škafar, Ljupka Dimitrovska, Janez Toplišek, Mojca Bervar, Matjaž Bunc, Marta Cvijić

Supplemental Table S1. Characteristics of the study group with concordant grading of MR and study group with discordant grading of MR

Supplemental Table S2. Characteristics of the study group who had intervention on mitral valve

Supplemental Table S3. Univariate Cox regression analysis for the composite end point

Supplemental Table S4. Multivariable Cox regression models for the composite clinical endpoint

Supplemental Figure S1. Study flow chart

Supplemental Figure S2. Forest plot showing hazard ratio (HR) and 95% confidence interval (CI) for composite outcome in our study group

Supplemental Figure S3. Prognostic value of quantitative parameters of MR severity, as a continuous variable.

Supplemental Table S1. Characteristics of the study group with concordant grading of MR (130 patients with EROA <40 mm^2 + RF <50% and 8 patients EROA ≥40 mm^2 + RF ≥50%) and study group with discordant grading of MR (46 patients with EROA <40 mm^2 + RF ≥50% and two patients with EROA ≥40 mm^2 + RF <50%).

	Concordant grading (n=138)	Discordant grading (n=48)	P-value
Atrial fibrillation (%)	32 (23)	18 (38)	0.332
LVEDD (mm)	63 ± 9	59 ± 12	**0.038**
LVESD (mm)	51 ± 11	49 ± 12	0.170
LVEDV index (ml)	105 (81-129)	86 (72-107)	**0.003**
LVESV index (ml)	66 (45-86)	58 (40-73)	0.086
LVEF (%)	37 (29-45)	35 (27-42)	0.242
Stroke volume index (ml/m^2)	32 ± 11	27 ± 9	**0.002**

Legend: LVEDD-left ventricular end-diastolic diameter, LVEDV-left ventricular end-diastolic volume, LVEF-left ventricular ejection fraction, LVESD-left ventricular end-systolic diameter, LVESV-left ventricular end-systolic volume.

Supplemental Table S2. Characteristics of the study group who had intervention on mitral valve.

	n=13 patients
LVEDV index (ml)	98 (82-140)
LVESV index (ml)	61 (39-84)
LVEF (%)	43 (33-50)
Stroke volume index (ml/m^2)	28 (25-38)
EROA (mm^2)	24 (18-41)
RegVol (ml)	52 (24-52)
RF (%)	39 (34-58)

Legend: EROA-effective regurgitant orifice area, RegVol-regurgitant volume, RF-regurgitant fraction, LVEDV-left ventricular end-diastolic volume, LVEF-left ventricular ejection fraction, LVESV left ventricular end-systolic volume.

Supplemental Table S3. Univariate Cox regression analysis for the composite end point.

	HR (95% CI)	P-value
EROA (mm^2)	1.039 (1.018-1.060)	**<0.001**
RegVol (ml)	1.013 (0.997-1.028)	0.105
RF (%)	1.018 (1.010-1.026)	**<0.001**

Legend: CI-confident interval, EROA-effective regurgitant orifice area, HR-hazard ratio, RegVol-regurgitant volume, RF-regurgitant fraction.

Supplemental Table S4. Multivariable Cox regression models for the composite clinical endpoint.

	Model 1		Model 2		Model 3	
	HR (95% CI)	P-value	HR (95% CI)	P-value	HR (95% CI)	P-value
Age (years)	1.038 (1.017-1.060)	<0.001	1.042 (1.020-1.065)	<0.001	1.036 (1.015-1.058)	0.001
LVESV index (ml)	1.006 (1.000-1.011)	0.037	1.005 (1.000-1.011)	0.055	1.008 (1.002-1.013)	0.008
SV index (ml/m²)	0.972 (0.957-0.994)	0.011	0.978 (0.957-1.000)	0.045	0.980 (0.959-1.001)	0.058
RV basal (mm)	1.593 (1.175-2.161)	0.003	1.537 (1.129-2.092)	0.006	1.609 (1.197-2.163)	0.002
PASP (mmHg)	1.006 (0.989-1.023)	0.497	1.005 (0.989-1.023)	0.532	1.008 (0.991-1.025)	0.378
RegVol (ml)	1.008 (0.992-1.023)	0.341				
EROA (mm²)			1.028 (1.006-1.051)	**0.014**		
RF (%)					1.016 (1.008-1.024)	**<0.001**

Legend: CI-confident interval, EROA-effective regurgitant orifice area, HR-hazard ratio, LVESV-left ventricular end-systolic volume, PASP-pulmonary artery systolic pressure; RegVol-regurgitant volume, RF-regurgitant fraction, RV-right ventricle, SV-stroke volume.

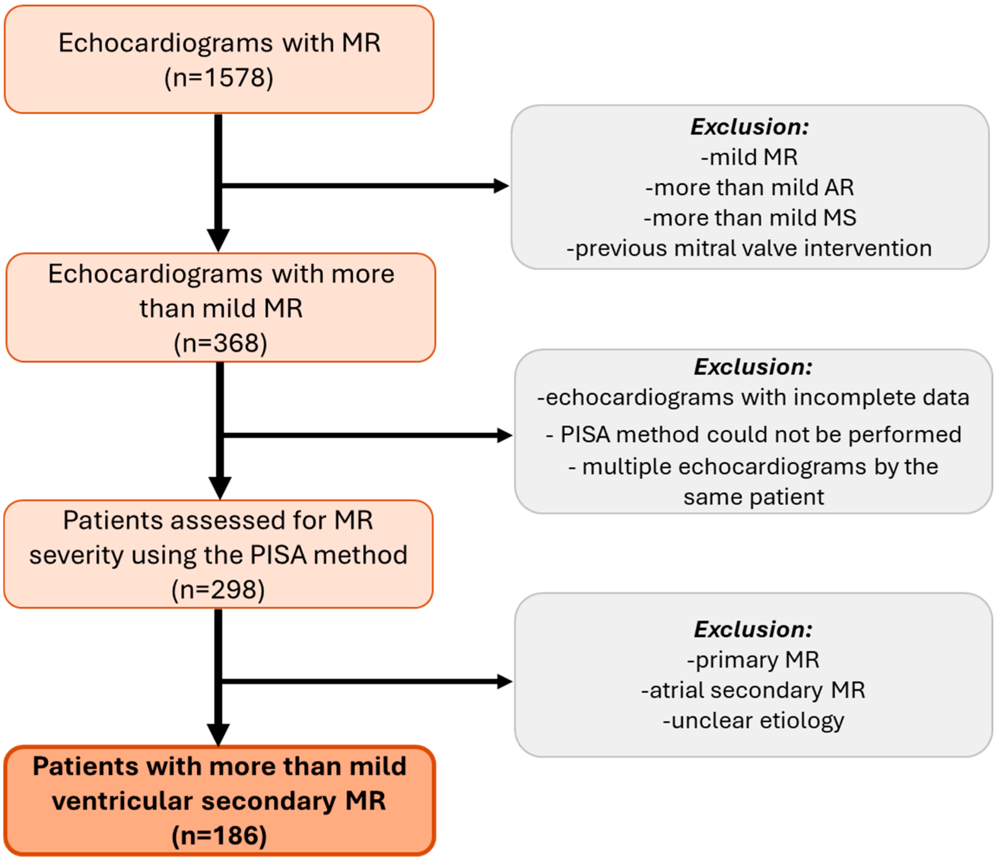

Supplemental Figure S1. Study flow chart.

Legend: AR-aortic regurgitation, PISA-proximal isovelocity surface area, MR-mitral regurgitation, MR-mitral stenosis.

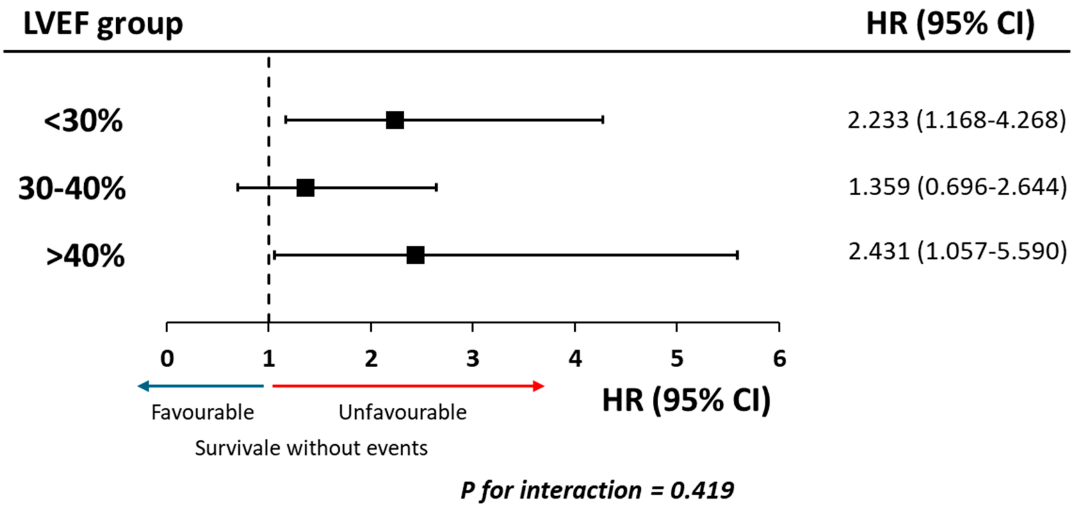

Supplemental Figure S2. Forest plot showing hazard ratio (HR) and 95% confidence interval (CI) for composite outcome in our study group.

Subgroup analyses show the impact of regurgitant fraction (RF ≥50%) versus RF <50% stratified by left ventricular ejection fraction (LVEF). No significant interaction was observed (p for interaction = 0.419), indicating a consistent prognostic effect of RF across LVEF subgroups (LVEF <30%, LVEF 30-40%, LVEF >40%).

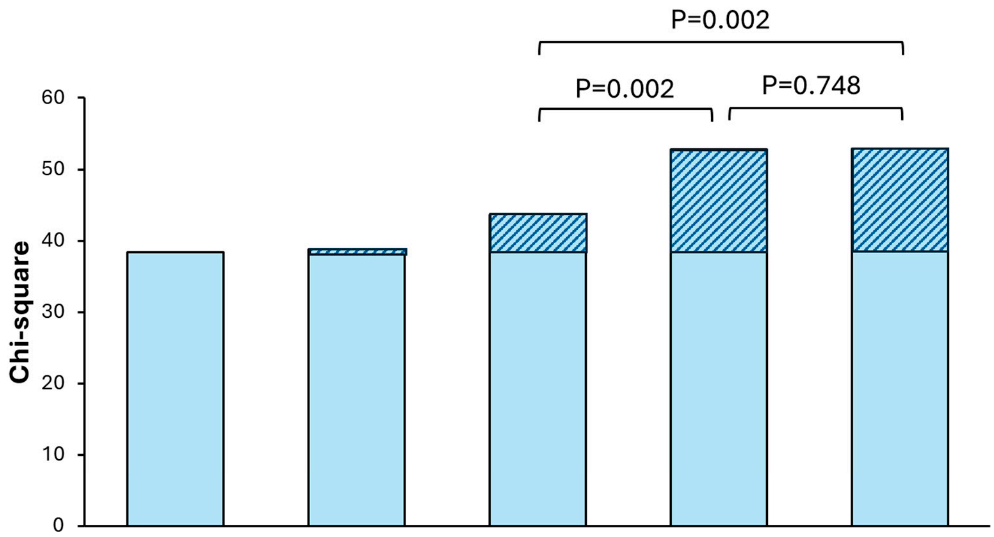

	Baseline model	Baseline model + RegVol	Baseline model + EROA	Baseline model + RF	Baseline model + EROA + RF
**P-value for comparison with baseline model**	-	0.497	0.023	<0.001	<0.001

Supplemental Figure S3. Prognostic value of quantitative parameters of MR severity, as a continuous variable.

Baseline model including age, LVESV index, SV index, RV diameter and pulmonary systolic pressure. Model with of RF and model with EROA were more accurate than baseline model in predicting risk of death and heart failure hospitalisation. No further significant incremental value was observed by adding EROA to the model with RF. The bar graphs show the chi-squares of each model; the marked part of each bar shows the change in chi-squares of the model compared to the baseline model. EROA-effective regurgitant orifice area, MR-mitral regurgitation, RegVol-regurgitant volume, RF-regurgitant fraction.
